# Exploring the Influence of Zinc Ions on the Conformational Stability and Activity of Protein Disulfide Isomerase

**DOI:** 10.3390/ijms25042095

**Published:** 2024-02-08

**Authors:** Ana Iochabel Soares Moretti, Viktoria E. Baksheeva, Andrei Yu. Roman, Tiphany Coralie De Bessa, François Devred, Hervé Kovacic, Philipp O. Tsvetkov

**Affiliations:** 1 Vascular Biology Laboratory (LIM64), School of Medicine, Heart Institute (InCor), Cardiopneumology Department, University of São Paulo, Campus Sao Paulo, Sao Paulo 05403-000, Brazil; 2 Aix Marseille Univ, CNRS, UMR 7051, INP, Inst Neurophysiopathol, Fac Sciences Médicales et Paramédicales, 13005 Marseille, Francefrancois.devred@univ-amu.fr (F.D.); herve.kovacic@univ-amu.fr (H.K.)

**Keywords:** neurophysiopathology, thiol proteins, protein disulfide isomerase, zinc binding

## Abstract

The interplay between metal ion binding and the activity of thiol proteins, particularly within the protein disulfide isomerase family, remains an area of active investigation due to the critical role that these proteins play in many vital processes. This research investigates the interaction between recombinant human PDIA1 and zinc ions, focusing on the subsequent implications for PDIA1’s conformational stability and enzymatic activity. Employing isothermal titration calorimetry and differential scanning calorimetry, we systematically compared the zinc binding capabilities of both oxidized and reduced forms of PDIA1 and assessed the structural consequences of this interaction. Our results demonstrate that PDIA1 can bind zinc both in reduced and oxidized states, but with significantly different stoichiometry and more pronounced conformational effects in the reduced form of PDIA1. Furthermore, zinc binding was observed to inhibit the catalytic activity of reduced-PDIA1, likely due to induced alterations in its conformation. These findings unveil a potential regulatory mechanism in PDIA1, wherein metal ion binding under reductive conditions modulates its activity. Our study highlights the potential role of zinc in regulating the catalytic function of PDIA1 through conformational modulation, suggesting a nuanced interplay between metal binding and protein stability in the broader context of cellular redox regulation.

## 1. Introduction

Protein disulfide isomerase (PDI, also known as PDIA1 or P4HB) is a widely expressed dithiol-disulfide oxidoreductase chaperone found predominantly in the endoplasmic reticulum (ER) [1,2]. It is essential for protein folding within the ER by catalyzing the formation, reconfiguration, and cleavage of disulfide bonds in cysteine residues, which is crucial for maintaining protein structure. PDI is sensitive to changes in the cellular redox environment, including oxidative stress, which is common under pathological conditions, including some proteinopathies such as Alzheimer’s and Parkinson’s Diseases (AD and PD), wherein misfolded proteins form insoluble aggregates. Substantial evidence indicates that PDI is upregulated in PD, Amyotrophic Lateral Sclerosis (ALS), and Huntington’s Disease [3], potentially serving a protective function in these conditions [4]. Due to PDI’s significant function in protein folding and ER-associated stress, it has been identified as a promising therapeutic target for treating neurodegenerative diseases. Modulating PDI’s function could therefore influence protein aggregation processes, ER stress levels, and ultimately, neuron survival.

**Figure 1:**
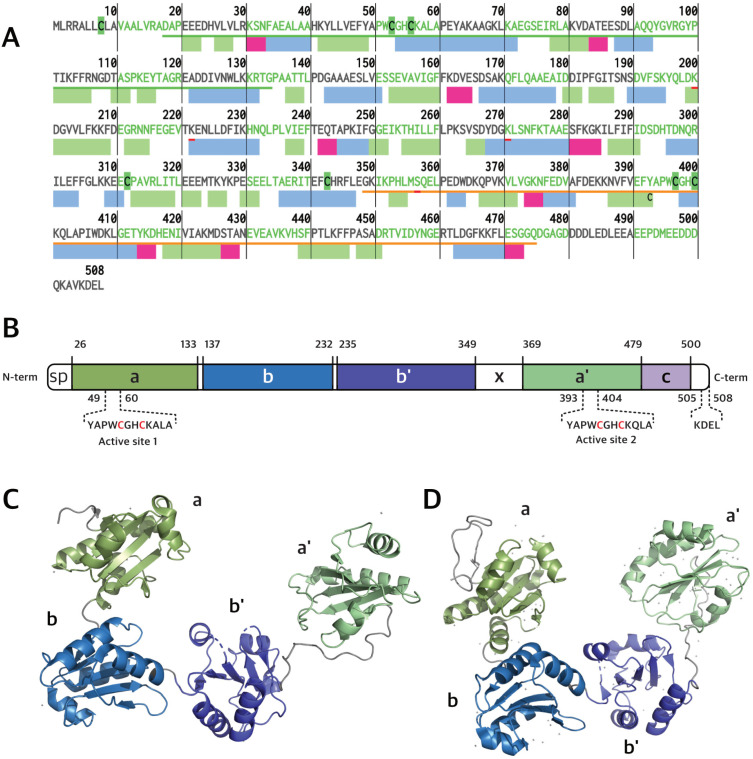
(**A**) Primary and secondary structure of human PDI. β-strands, α-helices, and turns are represented by rectangles colored green, blue, and magenta, respectively. (**B**) hPDIA1 domain organization showing its five domains (***a***, ***b***, ***b′***, ***a′***, ***c***) and linker (***x***) (Uniprot ID: P07237). Residues 1–18 correspond to signal peptide (SP). PDIA1 tertiary structures of oxidized (**C**) and reduced (**D**) forms based on PDB entry 4EL1 and 4EKZ, respectively.

PDIA1 is the prototype member of the PDI subfamily of thioredoxin superfamily proteins. Its sequence contains 508 a.a. residues (55 kDa, Figure 1A,C) forming a “U” shape structure composed of four sequential thioredoxin domains, named ***a***, ***b***, ***b′*** and ***a′*** [5,6,7,8,9,10] (Figure 1B). The **a** and ***a′*** domains at the arms of the “U” contain one CGHC redox-active motif each, while the ***b*** and ***b′*** domains at the bottom of the “U” are rich in hydrophobic residues involved in substrate binding [5,6,8,10,11]. Additionally, PDIA1 has a flexible x-linker motif between the ***b′*** and ***a′*** domains and a C-terminal tail rich in acidic residues, with a KDEL (Lys-Asp-Glu-Leu) ER retrieval motif [5,8,11]. The dithiol redox-active motifs endow PDIA1 with the ability to oxidize, reduce and isomerize disulfide bonds in target substrates. In addition, PDIA1 exerts chaperone activity, which is not directly dependent on the redox motifs [2,5,8]. PDIA1 activity is critical for redox proteostasis, and its loss of function contributes to proteotoxic diseases, as in the case of ALS [12,13] and AD [14]. It is also co-localized with FUS and TDP-43 inclusions in ALS and tau neurofibrillary tangles in AD [15]. Beyond its principal duties in redox protein folding and quality control within the ER lumen, PDIA1 also engages in additional roles. It interacts with the beta subunit of the prolyl-4 hydroxylase heterodimer and with the microsomal triglyceride transfer protein (MTTP), both of which are key enzymes in the processes of collagen hydroxylation and lipid transfer among liver membrane vesicles, respectively [10].

In addition, mounting evidence indicates that PDIA1 may exert several activities outside the ER, related to thiol redox signaling [16,17,18]. Proposed extra-ER locations of PDIA1 include the nucleus [19], cytoplasm [18,20,21] and, in particular, the membrane surface and extracellular milieu [22]. Extracellularly, PDIA1 is involved in thrombus formation [23,24,25], virus internalization [26], and support of vascular caliber remodeling during repair [22]. In most such effects, the main PDIA1-dependent redox activity is as a thiol reductase. For example, PDIA1 reduces critical cysteines from TNFR1/R2 on leukemia B-cells [27] and CD4-bound HIV envelope glycoprotein gp120 [28]. However, isomerase activity has been reported for the cell-surface regulation of ADAM-17 activity [28,29]. Also, extracellular PDIA1 can oxidize β1-integrins in vascular smooth muscle cells in association with the redox-dependent organization of cellular mechano-adaptation [30]. Additionally, PDIA1 regulates NADPH oxidase-dependent ROS generation associated with cell migration [20], Akt phosphorylation [22,31], and colon tumor cell aggressive phenotype [21]. Combined with redox-chaperone multifunctional versatility, the likely ability to translocate among cell compartments subserves PDIA1 in a number of (patho)physiological processes affected/monitored by thiol/disulfide or chaperone activity. In addition to its essential role in proteostasis and redox signaling, human PDIA1 can bind, transport and store hormones [32], vitamins [33], and lipids [34].

Metals are a particular type of ligand for thiol proteins in general. PDI has been reported to bind calcium [35], copper [36], and specifically zinc [37,38], an essential regulator of the activity and structure of potentially >3000 proteins [39,40] and an important redox modulator as well [41]. The effects of zinc binding on PDIA1 function, however, have been investigated mainly in a context reminiscent of proteostasis, in which zinc associates with loss of—or interference with—PDIA1 isomerase activity [37,38], oligomerization [37] and impaired ER-dependent protein folding [38]. Intracellular concentrations of free zinc are not well established since their detection highly depends on the methods used [39,40]. However, there is an emerging consensus that in most subcellular compartments, they are in the picomolar to low nanomolar range [39]. Zinc levels can potentially exhibit substantial variability within distinct organelles, including the ER, due to the presence of specific transporters and interactions with other ions [42]. Neurons in particular seem exposed to high concentrations of extracellular zinc [42]. In such cases, the relative ratios of zinc to PDIA1 levels may be lower than those previously assumed. Thus, given the context of broader PDIA1 signaling effects listed above, it is important to expand the investigation of zinc effects on PDIA1. In this study, while focusing on the impact of zinc on PDI structural stability and its reductase activity, we uncovered the capacity of zinc ions to modulate the reductase activity of PDIA1.

## 2. Results

### 2.1. Oxidation of PDIA1 Reduces the Stoichiometry of Zinc Binding

**Figure 2:**
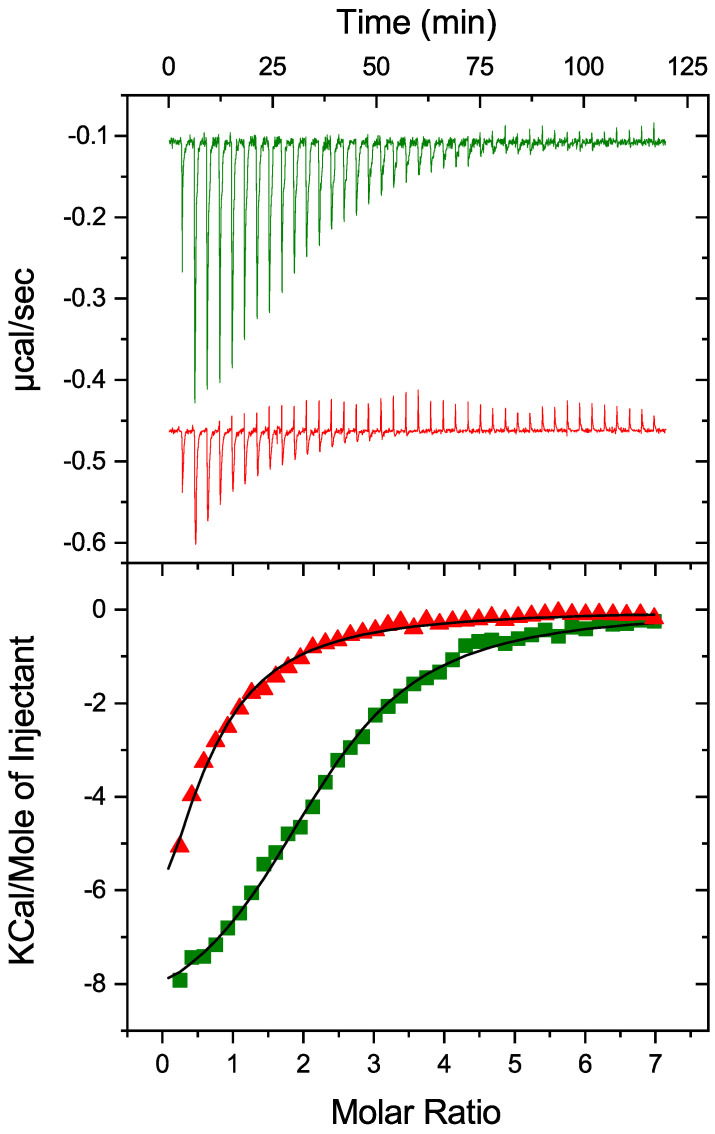
Impact of PDIA1 redox state on thermodynamic parameters of its interaction with Zn^2+^. Oxidized (red) and reduced (green) hPDIA1 were titrated by ZnCl_2_ solution in 50 mM Tris buffer, pH 7.3 at 25 °C using isothermal titration calorimetry. Top panel represents corresponding ITC titration curves, bottom panel represents binding curves with best fit using one-set-of-sites binding model.

To investigate the potential impact of PDIA1 redox state on thermodynamic parameters of its interaction with Zn^2+^, oxidized and reduced-PDIA1 were titrated by ZnCl_2_ solution using isothermal titration calorimetry (Figure 2). Both redox forms were able to bind Zn^2+^ with relatively close association constants but significantly different stoichiometry (Table 1). PDIA1 in reduced state binds Zn^2+^ with association constant of 0.9 ± 0.1 × 10^5^ M^−1^, and a stoichiometry of 2.3 ± 0.1. Oxidation of PDIA1 led to a slight decrease in association constant but to a significant decrease in the stoichiometry till 0.5. While both reduced and oxidized states of PDIA1 are able to bind zinc, the changes observed in the binding parameters reveal that zinc may have a different effect on the conformation of PDI depending on its redox state.

### 2.2. PDIA1 Thermal Denaturation in Different Redox States

**Figure 3:**
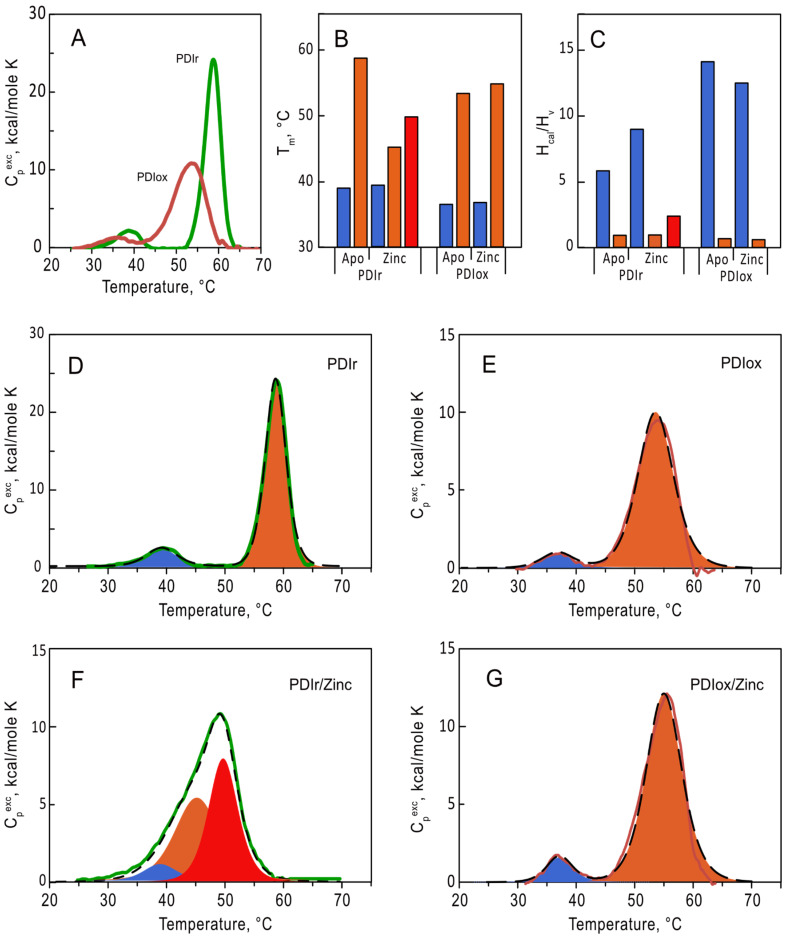
Analysis of thermal denaturation of oxidized (red line) and reduced (green line) forms of PDIA1 at 2 mg/mL (36 µM) in 50 mM Tris, pH 7.0 using DSC. (**A**) Temperature dependence of excess heat capacity of oxidized and reduced forms. (**B**,**C**) Temperatures (T_m_) and the ratio of va not Hoff to calorimetric enthalpies (∆H_vH_/∆H_cal_) of the first (blue), second (orange), and third (red) denaturation transitions of oxPDIA1 and redPDIA1 in the absence and in the presence of 10-fold excess of zinc ions. (**D**–**G**) Deconvolution of denaturation transitions, respectively for redPDIA1 and oxPDIA1 in the absence and in the presence of zinc ions. Deconvolution peaks are shown as filled areas (blue, orange and red for the first, second, and third transitions), fitting curves are shown in black dashed curves.

To evaluate the impact of zinc on conformation of PDIA1 in different redox states (oxidized and reduced) we first studied the thermal stability of apo forms of PDIA1 using differential scanning calorimetry (DSC) (Figure 3). Without zinc both redox states of PDIA1 denatured in two pronounced transitions (Figure 3A). The temperatures of the first transition were equal to 36.5 °C and 39.3 °C for oxidized and reduced forms of PDIA1, respectively, and those of the second transition were 53.3 °C and 58.7 °C (Figure 3B, Table 2). The presence of two peaks on the protein denaturation profile generally corresponds to the denaturation of two separate folding domains, which seems reasonable in the case of PDIA1, considering its domain structure. Interestingly, the fitting of denaturation curves of both oxidized and reduced-PDIA1 with “non-two-state” model revealed that the ratios of va not Hoff enthalpy (∆H_vH_) to calorimetric enthalpy (∆H_cal_) for the first transition are significantly higher than 1 (Figure 3C, Table 2). This parameter generally reflects the number of folding units (domains) denatured in the peak.

### 2.3. Impact of Zinc Ions on Thermal Denaturation of PDIA1

To investigate the effect of Zn^2+^ binding on thermal stability of PDIA1, we followed denaturation of oxidized and reduced-PDIA1 in the presence of 10-fold excess of Zn^2+^ using DSC (Figure 3F,G). The presence of zinc did not significantly impact the denaturation profile of oxidized PDIA1 (Figure 3E,G). In contrast, the denaturation profile of reduced-PDIA1 underwent major changes upon the addition of Zn^2+^ (Figure 3D). Instead of two pronounced peaks on denaturation curves of the protein, only one peak was observed in the presence of Zn^2+^. Still, deconvolution of the peak with the “non-two-state” model disclosed three denaturation transitions with temperatures 39, 45 and 50 °C (Figure 3B,D). The ratios of va not Hoff and calorimetric enthalpy (∆H_vH_/∆H_cal_) did not change dramatically for the first low-temperature denaturation peak in the presence of zinc (Figure 3C). In contrast, the high-temperature denaturation peak, with ∆H_vH_/∆H_cal_ ratio close to one in the absence of zinc, was split into two transitions with higher ∆H_vH_/∆H_cal_ ratio (Figure 3C,D, Table 2) in the presence of zinc, indicating a significant rearrangement in interdomain interaction of reduced-PDIA1 upon zinc binding. Thus, our data clearly demonstrate that zinc can modulate the folding stability and tertiary structure of reduced-PDIA1 but not impact on the protein when it is in an oxidized state.

### 2.4. Effect of Zinc on PDIA1 Activity

**Figure 4:**
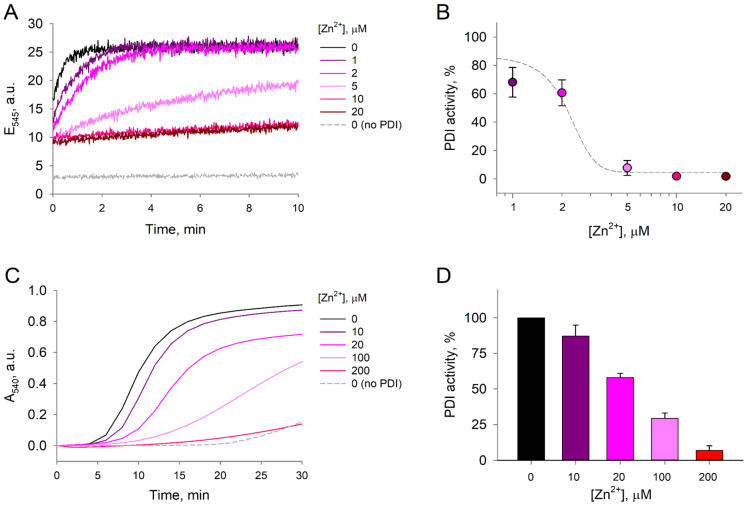
Thiol-reductase activity of PDI. The effect of Zn^2+^ on PDI activity was measured in the Di-E-GSSG reductase assay and insulin precipitation turbidity assay. (**A**) Representative fluorescence emission curves of 1 µM Di-E-GSSG at 545 nm measured over 10 min in the presence of 1 µM redPDI and 1–20 µM Zn^2+^. The reduction of 1 µM Di-E-GSSG by 5 µM DTT served as a negative control. Zinc without PDIA1 does not react with Di-E-GSSG. (**B**) Comparative PDI activity in the presence of increasing Zn^2+^ concentrations estimated from the linear phase of Di-E-GSSG fluorescence emission build-up. Each data point was plotted based on three independent measurements. Estimated dose–response fit is presented as a dashed line. (**C**) Representative curves of 1 mg/mL insulin absorbance at 540 nm in the presence of 5 µM redPDI and 10–200 µM Zn^2+^. The precipitation of insulin by 10 mM DTT served as a negative control. (**D**) Comparative PDI activity in the presence of increasing Zn^2+^ concentrations estimated from the linear phase of insulin precipitation. Each data point was plotted based on three independent measurements.

Considering that Zn^2+^ binds to reduced-PDIA1 and causes major changes of its denaturation profiles, which are associated with significant conformational changes in protein structure, we hypothesized that Zn^2+^ binding could influence activity of PDIA1 in reduced state. To test this hypothesis, we first assessed reductase activity through the di-eosin-glutathione disulfide (Di-E-GSSG) assay, which is based on the ability of reduced-PDIA1, exposed or not to Zn^2+^, to reduce a probe composed of glutathione disulfide (GSSG) labeled with two molecules of eosin [43]. A suppression in Di-E-GSSG cleavage was observed after exposure of reduced-PDIA1 (1 µM) to Zn^2+^ from 1 to 20 µM concentrations (Figure 4A). Di-E-GSSG turnover decreased by ~32% already at 1 µM Zn^2+^ and by >90% at 5 µM (Figure 4B). EC50 of Zn^2+^ was assessed using sigmoidal dose response model fit from SigmaPlot 12.0 (Systat Software Inc., San Jose, CA, USA) and was estimated to be 2.2 ± 0.2 µM. Different concentrations of Zn^2+^ did not affect the fluorescence of Di-E-GSSG in the presence of oxidized PDIA1, which had no reductase activity since the cysteines in the active site were forming a disulfide bond. These results suggest that reduced-PDIA1 incubation with Zn^2+^ inhibits PDIA1 reductase activity, which is in good agreement with our DSC data.

Since different assays of PDIA1 address distinct aspects of enzyme function and structure, we also performed a second assay to investigate reduced-PDIA1 activity using the classical insulin turbidimetric method. We observed that zinc inhibited reduced-PDIA1 (5 μM) activity in a dose-dependent manner (Figure 4C). At 20 µM, Zn^2+^ inhibited approximately 40% of reduced-PDIA1 activity, while at 200 µM Zn^2+^ inhibited more than 90% of reduced-PDIA1 activity (Figure 4D). It should be noted that at Zn^2+^ concentrations exceeding 500 µM spontaneous (PDIA1-independent) insulin aggregation was observed, which is in agreement with previous observations [44,45]. However, no such aggregation was detectable at Zn^2+^ concentrations below 100 µM, and at 100–200 µM only 5–10% of insulin precipitated in a PDIA1-independent manner.

Overall, our findings reveal that PDIA1 binds zinc ions in a redox-dependent manner. Zinc binding to reduced PDIA1 form triggers pronounced conformational changes, which in turn influence PDIA1 catalytic activity.

## 3. Discussion

### 3.1. Stoichiometry of Zinc Binding to PDIA1

Zinc binding to proteins from the PDI subfamily has been reported in several publications [37,46]. In 2004, zinc binding to rat PDI was studied using a gel-filtration method. Authors reported a different zinc stoichiometry for PDIA1 dimers and monomers of 1 and 0.7, respectively. However, these values were only estimates, given the limited accuracy of the methodology used and the absence of control for PDI redox state. The authors also demonstrated that zinc promotes formation of PDIA1 dimers, which is in line with our findings. It should be noted that PDIA1 can also form disulfide-independent dimers without zinc ions [47], and since dimerization is an equilibrium process, both monomers and dimers could be present in cells and play different roles [47]. In this context, the regulatory role of zinc as a modulator of PDIA1 activity, which depends on its dimerization, could be considered.

Recently, thermodynamic parameters of zinc binding to ERp44 (PDIA10) were obtained by ITC, and the crystal structure of the complex was determined with a 2.45 Å resolution [46]. Interestingly, while crystal structure showed the presence of five zinc ions located in the dimer interface of ERp44 (or 2.5 zinc ions per monomer), ITC clearly demonstrated stoichiometry of 1.5 for zinc binding to ERp44 probably due to the small enthalpy of zinc binding to one of the sites [46]. The stoichiometry we found from ITC for zinc binding to PDIA1 in a reduced state is 2.3, which is higher than for ERp44, but very close to the 2.5 found from the crystal structure. ERp44 differs from PDIA1 not only by the lack of an **a′** domain but also by the presence of a conserved histidine cluster. Nevertheless, these results allow us to hypothesize that, like in case of ERp44 [46], binding of one of the zinc ions to PDIA1 could also occur at the dimer interface [37]. Together with the 0.5 stoichiometry for oxidized PDIA1, these findings suggest that while one zinc ion binds to the dimer interface of the PDIA1 the others interact with Cys residues located in the **a** and **a′** domains. Indeed, the other most common zinc chelators, His, Glu, and Asp a.a, are not impacted by oxidation, thus making Cys residues responsible for the loss of binding of two additional zinc ions to oxidized PDIA1 [37]. This loss could lead in turn to the absence in conformational changes of oxidized PDIA1 upon zinc increase, as observed in the case of reduced PDIA1. Another potential explanation for the decrease in stoichiometry upon PDIA1 oxidation could be linked with the differences in conformation of reduced and oxidized states, which is in line with our DSC experiments as well as other crystallographic [9] and computational [5,7] data.

### 3.2. Conformational Differences between Oxidized and Reduced PDIA1

The thermal denaturation of PDIA1 in both redox states revealed the existence of two distinct denaturation peaks (Figure 3A, Table 2). It should be noted that denaturation of oxidized PDIA1 occurs at a much lower temperature than denaturation of its reduced state, indicating that PDIA1 oxidation dramatically impacts its conformation. The decrease in thermostability in an oxidized state is common but not obligatory for proteins [48]. Our result is in agreement with a recent study conducted through CD [11]. Even though the authors did point out the biphasic nature of some of their denaturation curves, they did not use it in their final analysis. This explains why they reported single-wide transitions for reduced and oxidized PDIA1, with temperature midpoints (54 and 48–50 °C, respectively) falling between the two transitions we found using DSC. Revising their data using a two-transitions model, we found the temperatures of both transitions found by CD are close to the temperatures we found by DSC. A small temperature shift of 2–3 °C between denaturation temperatures (T_m_) is often observed with CD and DSC methods [49,50,51] because the latter reflects mainly denaturation of tertiary protein structure, while the former reflects mainly denaturation of secondary structure that could occur slightly later. The observed difference in T_m_ could also be explained by different buffer conditions (50 mM Tris used for DSC experiments and 20 mM Sodium Borate containing 100 mM NaCl used for CD measurements).

Furthermore, DSC allowed us to obtain calorimetric enthalpy (∆H_cal_) and va-not-Hoff enthalpy (∆H_vH_) for both transitions, thus giving access to the number of folding units denatured in each transition calculated from the ratio ∆H_vH_/∆H_cal_. The ratio ∆H_vH_/∆H_cal_ for the second denaturation peak of both reduced and oxidized PDIA1 is close to one. Considering the four domains of PDIA1, this suggests strong interdomain interactions, which lead to their denaturation as a single folding unit. In contrast, the ratio ∆H_vH_/∆H_cal_ for the first denaturation peak of reduced-PDIA1 is significantly higher than one. Taking into account the small ∆H_cal_ of this denaturation, it is difficult to explain this high ratio by desaturation of several domains within this peak. The denaturation curves obtained by Okumura et al. [1] for the ***xa′c*** fragment of PDIA1 allow us to hypothesize that this low-temperature peak corresponds to the denaturation of the **c** or **a′** domain. Indeed, even if the authors did not deconvolute the DSC curves and did not mention the existence of two denaturation peaks, the ***xa′c*** fragment denatures in two overlapping but still clearly distinct transitions at approximately 43 and 53 °C. Considering the elevated ratio of ∆H_vH_ to ∆H_cal_, it is plausible that the **c** domain of PDIA1 may exist in either a folded or unfolded state, as depicted in Figure 2 of reference [52], thus decreasing the overall ∆H_cal_ value of this peak and consequently increasing the ∆H_vH_/∆H_cal_ ratio. At last, for oxidized PDIA1, the ratio ∆H_vH_/∆H_cal_ is almost doubled, indicating significant changes in protein conformation that are consistent with findings by Wang et al. [2], who demonstrated that oxidation of PDIA1 releases the **a′** domain from compact conformation and activates PDIA1 chaperone activity.

### 3.3. Impact of Zinc on PDIA1 Conformation

Zinc binding to reduced PDIA1 led to significant changes in protein thermostability without modification of the overall enthalpy of denaturation (Table 1). Indeed, while the thermodynamic parameters of the low-temperature transition were not impacted in the presence of zinc ions, the denaturation of the second transition of reduced-PDIA1 was significantly affected. Being a single denaturation unit in the absence of zinc with T_m_ equal to 58.7 °C, PDIA1 denaturation splits into two distinct transitions, with lower T_m_ equal to 45.3 and 49.8 °C in the presence of zinc. This implies that in the absence of zinc, the domains (most probably ***a***, ***b***, ***b′*** and ***a′***) exhibited a strong interaction and thus denatured as a single folding unit. However, in the presence of zinc, this interaction was lost, leading to separate domain denaturation, which presumably increases the protein’s flexibility. This conclusion is supported by the values of ratio ∆H_vH_/∆H_cal_ for splitted transitions, which in sum gives four, which corresponds to four domains ***a***, ***b***, ***b′*** and ***a′***. Thus, considering the values of the ratio ∆H_vH_/∆H_cal_ and protein structure, it is reasonable to hypothesize that one of the pleated peaks correspond to denaturation of domains ***b*** and ***b′*** still interacting, while domains ***a*** and ***a′*** denaturate in another peak independently (∆H_vH_/∆H_cal_ equal to 2.5). It should be noted that while the destabilization of protein structure upon zinc binding is a rather common phenomenon observed previously for proteins with different structures [53,54] and could be explained by different mechanisms, it seems that in the case of PDIA1, this destabilization is most probably caused by the breaking of inter-domain interactions.

While the reduced form of PDIA1 undergoes significant conformational changes in the presence of zinc, the influence of zinc on the oxidized form of PDIA1 is relatively minor. This is likely attributable to the lack of zinc binding at two additional sites, which would be accessible only in the reduced state and would be responsible for substantial rearrangements in domain interactions. Conversely, the binding of zinc at the dimer interface in the oxidized form would not result in similar effects.

### 3.4. Zinc Impact on PDIA1 Activity

Zinc, a vital micronutrient, plays a key role in preserving the structural integrity and functionality of numerous proteins, including enzymes, transcription factors, receptors, and more [40,53,55,56,57,58]. Under normal physiological conditions, the concentration of zinc is meticulously regulated and maintained in the cytoplasm within the subnanomolar range, primarily by cysteine-rich metallothionein proteins. However, oxidative stress can provoke significant an increase in free zinc levels, as the ion-binding sulfhydryl groups on metallothioneins are susceptible to oxidation [59]. Under such oxidative conditions, proteins that have a moderate affinity for zinc and experience substantial conformational alterations upon zinc binding, like PDIA1, may serve as initial sensors and mediators in the cellular response. This observation aligns with our prior research on a zinc-sensitive signaling protein, where we found that zinc enhances redox sensitivity, leading to the formation of a conformer that exhibits an altered functional state [58]. A delayed or insufficient response to a prolonged rise in zinc concentrations may contribute to the advancement of neurodegenerative conditions such as AD, PD, ALS, etc. [60]. Such pathologies are marked by the zinc-mediated aggregation of proteins like tau [61], beta-amyloid [62], and TDP-43 [54], and are also characterized by weak affinity to zinc ions.

If PDIA1 function is affected by zinc-binding, this could provide a deeper understanding on the role of this enzyme in antioxidant response. Indeed, earlier, it was demonstrated that zinc is able to reduce isomerase activity of PDIA1 [37]. The functional tests used in our study also showed that PDIA1 activity is modified in the presence of zinc. Interestingly, it has been recently demonstrated that zinc-loaded flavonoids (e.g., rutin and quercetin) are significantly more effective in PDI inhibition than their respective apo-forms [63]. Although the observed effect was initially attributed to the complex of the flavonoid molecule with zinc, our findings suggest that the formation of the Zn^2+^-PDIA1 complex is also a viable factor and should not be overlooked. Indeed, the referenced study did not include free zinc as a control in the sample. Furthermore, the association constants of flavonoid-Zn^2+^ are estimated to be similar to or lower than the PDIA1-Zn^2+^ constants, as determined in our study. This suggests that the zinc ion could indeed be transferred to PDIA1 and provoke its inhibition.

While zinc itself lacks redox properties, it can respond to changes in environmental redox homeostasis, functioning either as an antioxidant or as a redox-dependent regulator of protein activity [41,58,64]. Indeed, zinc can bind to sulfhydryl groups, thereby preventing their oxidation and maintaining the target protein in its reduced state [65,66]. Our research uncovers a potential regulatory mechanism wherein zinc binding to reduced PDIA1 inhibits its reductase activity. The observed moderate zinc binding constant for PDIA1 suggests its function as a regulatory element rather than a structural one. This is based on the understanding that a higher binding constant, which is characteristic of zinc-finger proteins, would confer greater stability to the zinc-PDIA1 complex, thereby restricting its ability to undergo functional transitions in response to fluctuations in zinc concentration, particularly during zinc waves where zinc levels can transiently rise to low micromolar concentrations.

Overall, our findings, in conjunction with previous reports in the literature [37,38,66], indicate that the inhibition of PDIA1 activity by zinc is contingent upon its redox state and conformational changes. This aspect is likely to be crucial in regulating the redox signaling effects of PDIA1, which occur in various subcellular localizations and predominantly involve PDIA1’s reductase activity. Given the typically lower concentration of free zinc ions in the ER, it seems less probable that zinc significantly influences PDIA1 activity in this context. Instead, zinc’s impact on PDIA1’s reductase function might be more pronounced in other reductive subcellular environments where zinc concentrations are substantially higher and subject to fluctuations, such as zinc waves. This points to a more targeted regulatory role for zinc in specific cellular compartments.

## 4. Materials and Methods

### 4.1. Protein Purification

Human protein disulfide isomerase was produced and purified as described [67]. peT28a vector (Novagen, Madison, WI, USA) was expressed in *E. coli* strain BL21 (DE3) codon plus grown in medium Terrific broth (yeast extract 24 g/L, tryptone 20 g/L, glycerol 4 mL/L, 0.17 M KH_2_PO_4_ and 0.72 M K_2_HPO_4_) at 37 °C. When the apparent absorbance of the culture at 600 nm reached 0.6, protein expression was induced with 0.1 mM IPTG for 6 h. The bacterial pellet was recovered after centrifugation at 10,000 rpm for 15 min and resuspended in the resin equilibration buffer and wash buffer (NaCl 300 mM and Tris 50 mM, pH 7.5). Protein was extracted from the bacterial lysate by adding lysozyme (2.0 mg/mL) to the equilibration/washing buffer, DNAsase (5 μg/mL) and PMSF (0.5 mM), then the lysate was incubated on ice for 2 h under orbital agitation followed by centrifugation at 12,000 rpm to obtain the supernatant. PDIA1 was finally purified from the lysate by immobilized metal affinity resin (TALON affinity resin) according to the manufacturer’s instructions for native proteins. After protein purification, samples were dialyzed against an equilibration buffer (Tris 50 mM, NaCl 150 mM) to remove imidazole. ReducedPDIA1 (redPDIA1) was obtained by treating the protein with excess 20 mM DTT, followed by the removal of excess DTT by overnight dialysis and buffer exchange using a FPLC desalting column. To obtain oxidized PDIA1 (oxiPDIA1), the enzyme was treated with 10–20 mM hydrogen peroxide, and after 2 h, excess hydrogen peroxide was removed with a FPLC desalting column. When required, the protein was concentrated by ultrafiltration (Amicon Ultra, cut-off 50 kDa, Millipore (Burlington, MA, USA)). The PDIA1 used in all experiments was fully active with respect to reductase activity [68]. The residue numbering employed was for human PDIA1 with the signal sequence. Our clone, however, contained the human PDIA1 sequence without amino acids 1–17 corresponding to the peptide signal. Thus, this protein has only six internal cysteine residues, given that cys8 is not present.

### 4.2. Detection of Free Protein Thiols by the DTNB Assay

The redox status of PDIA1 was confirmed by the DTNB reaction as follows. Two milligrams per ml of PDIA1 were oxidized or reduced as described above using 20 mM H_2_O_2_ or 20 mM DTT, respectively. DTT and hydrogen peroxide were removed by size-exclusion chromatography in a Sephadex G-25 column (GE HealthCare (Chicago, IL, USA)) equilibrated with resin equilibration buffer and wash buffer (300 mM NaCl and 50 mM Na_2_HPO_4,_ pH 7.0). The protein amount was diluted to a final concentration of 10 µM and assayed for free thiol quantification by Ellmans’ reagent (DTNB). DTNB is reduced stoichiometrically by free thiols by an exchange reaction, forming a mixed disulfide with protein and a yellow product, TNB. PDIA1 was diluted ten-fold in 100 µM tris buffer (pH 8.0) containing 1% SDS and 10 mM DTNB (prepared in sodium phosphate buffer pH 8.0). Samples were incubated for 15 min at room temperature, and absorbance was measured at 412 nm. DO_412_ was divided by the DTNB molar extinction coefficient (13,600 M^−1^/cm^−1^). Samples were assayed in triplicate for all six independent experiments, and the results are expressed as the mean ± SEM. Significance was analyzed by one-way ANOVA with Bonferroni’s post hoc test. PDIA1 in the reduced state showed a robust amount of free thiols (110.15 ± 16.22 µM), while the oxidized form (6.45 ± 2.83 µM) had about 5% of total free thiols observed with reduced PDIA1 (Figure S1).

### 4.3. Isothermal Titration Calorimetry (ITC)

Thermodynamic parameters of zinc binding to PDIA1 were measured using a MicroCal iTC_200_ instrument (MicroCal, Northampton, MA, USA, now part of Malvern Instruments Ltd., (Malvern, UK)) as described previously [53]. Experiments were carried out in a 50 mM Tris buffer, pH 7.3 at 25 °C. Aliquots of ZnCl_2_ solution (2 μL) were injected into the 200 μL cell-containing protein solution to achieve a complete binding isotherm. The PDIA1 concentration in the cell was 33 μM, and the ZnCl_2_ concentration in the syringe was 1 mM. The heat of dilution was measured by injecting the ligand (ZnCl_2_) into the buffer solution; the values obtained were subtracted from the heat of reaction to obtain the effective heat of binding. The resulting titration curves were fitted using MicroCal Origin 7.0 software with a “one-set-of-sites” model. The association constant (*K_a_*), enthalpy of interaction (∆*H*) and binding stoichiometry (*N*) were determined by a non-linear regression fitting procedure.

### 4.4. Differential Scanning Calorimetry (DSC)

DSC thermograms of 2 mg/mL (36 µM) PDIA1 in 50 mM Tris buffer at pH 7.0 were obtained using a differential scanning microcalorimeter VP-DSC (MicroCal, Northampton, MA, USA, now part of Malvern Instruments Ltd., Malvern, UK) according to the manufacturer’s instructions. The sample was loaded into a 500 μL calorimetric cell. Scans were recorded from 15 °C to 90 °C with a heating rate of 1 K/min. The curves were corrected for the instrumental baseline obtained by heating the solvent used for protein solution. The reversibility of denaturation was checked routinely by sample reheating after cooling in the calorimetric cell. Thermograms were treated using Origin 7 software to obtain excess heat capacity curves (OriginLab Corporation, Northampton, MA, USA) as previously described [69], then data was fitted using the “non-two-state” denaturation model available in manufacture software to determine calorimetric (∆*H_cal_*) and Van’t Hoff (∆*H_vH_*) enthalpies as well as the temperature of denaturation (*T_m_*) for each individual transition.

### 4.5. Di-Eosin-Glutathione Disulfide (Di-E-GSSG) Assay

The reductase activity of redPDIA1 was assessed using Di-E-GSSG, as described previously [43]. The probe Di-E-GSSG was prepared by incubating 200 µM GSSG with 2 mM eosin isothiocyanate in 100 mM phosphate buffer [pH 8.8] containing 2 mM EDTA at room temperature overnight in the dark. The mixture was passed down a HiTrap desalting column (Cytiva, Marlborough, MA, USA), and different fractions were collected. A fold change of fluorescence (Ex: 520 nm, Em: 545 nm) was calculated using samples subjected to either buffer or 20 mM DTT. Fractions with a fold-change >6 were kept. The concentration of Di-E-GSSG was determined by using the molar absorption coefficient for eosin (ε = 56,000 M^−1^ cm^−1^) at 525 nm in 100 mM potassium phosphate [pH 8.8] containing 2 mM EDTA. Reduction of Di-E-GSSG was carried out by incubation of 1 µM redPDIA1 in assay buffer (Tris [pH 7.5] containing 0.1 M NaCl), 5 µM DTT and 1 µM of the Di-E-GSSG probe in the presence or absence of Zn^2+^ (1 to 20 µM concentration). The increase in fluorescence was determined in a series of three independent experiments by monitoring excitation at 520 nm and emission at 545 nm in a Horiba Jobin Yvon FluoroMax 3 spectrofluorometer (Horiba, Kyoto, Japan) for 10 min with 1 s intervals. The reduction of 1 µM Di-E-GSSG by 5 µM DTT served as a negative control, and it was determined that zinc without PDIA1 does not react with Di-E-GSSG. The kinetics of Di-E-GSSG breakdown was assessed by plotting the linear regression trend of the initial portion of the fluorescence emission curve, using Sigmaplot 12.0 software (Systat Software Inc., San Jose, CA, USA). Data were normalized to the fluorescence of redPDIA1 in the absence of Zn^2+^ and represented as a percentage of reductase activity.

### 4.6. Insulin Precipitation Assay

The thiol redox activity of redPDIA1 was further assessed by the insulin reductase assay method as described previously [43]. PDIA1 reduction was carried out as described above in a protein purification method. An amount of 1 mg/mL of recombinant human insulin (Sigma-Aldrich (St. Louis, MO, USA)) was incubated for 30 min with 5 µM redPDIA1 in the presence or absence of Zn^2+^ (10 to 200 µM concentration). The precipitation reaction was initiated by adding 20 mM Tris buffer [pH 7.5] containing 150 mM NaCl and 10 mM DTT. PDIA1 reductase activity was monitored spectrophotometrically by measuring insulin precipitation at 540 nm. The reduction of insulin by 10 mM DTT served as a control. Three independent experiments were performed, and each sample was detected in triplicate. Data were normalized to the vehicle and represented as a percentage of reductase activity.

## Figures and Tables

**Table 1:** Thermodynamic parameters of zinc binding to PDI in a 50 mM Tris-HCl buffer (pH 7.3) at 25 °C.

PDI Forms	K_a_ M^−1^	∆H kcal/mol	∆S cal/mol K	N
Reduced	(9.0 ± 0.5) × 10^4^	−9.1 ± 0.2	−8.01	2.3 ± 0.1
Oxidized	(4.4 ± 0.4) × 10^4^	−14.0 ± 2.2	−25.5	0.5 ± 0.1

**Table 2:** Parameters of thermal denaturation of PDI at a different level of oxidation.

Oxidative State	Ion	Peak	T_m_	∆H_cal_	∆H_vH_	∆H_vH_/∆H_cal_
Reduced		1	39.3	17.0	110	6.5
		2	58.7	124.0	190	1.5
Oxidized		1	36.5	7.6	105	13.8
		2	53.3	83.3	104	1.2
Reduced	Zn^2+^	1	39.0	10.2	88	8.8
		2	45.3	54.1	81	1.5
		3	49.8	52.9	125	2.4
Oxidized	Zn^2+^	1	36.8	10.3	129	12.5
		2	54.6	102.0	105	1.0

## Data Availability

Data contained within the article.

## Supplementary Materials

The following supporting information can be downloaded at: https://www.mdpi.com/article/10.3390/ijms25042095/s1.
