# Supplementary material for: Exploring the Influence of Zinc Ions on the Conformational Stability and Activity of Protein Disulfide Isomerase
Source: Int J Mol Sci. 2024 Feb 8;25(4):2095. doi: 10.3390/ijms25042095 (PMC10889200; doi:10.3390/ijms25042095)
Supplement: Supplementary file 1 [file ijms-25-02095-s001.zip › ijms-2770463-supplementary.pdf]

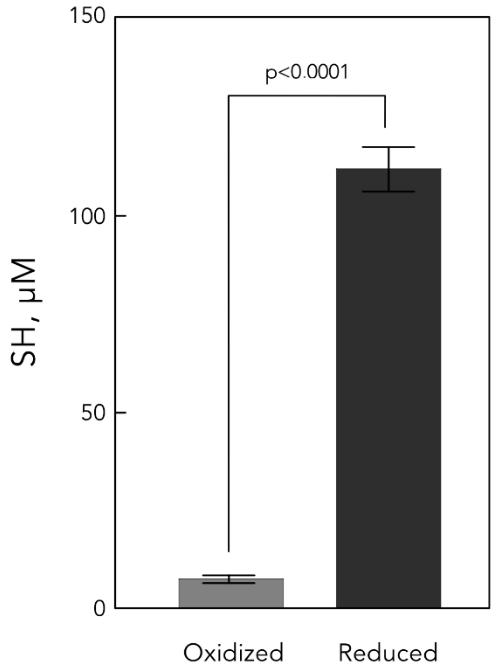

Figure S1: Analysis of free thiols on PDI in distinct redox states. DTNB measurements were used to assess free thiols on the oxidized and reduced forms of 20 μM PDIA1. Six independent experiments were performed in triplicate. Data represent mean ± SEM; *n*=6.
